# The Immunogenicity and Immune Tolerance of Pluripotent Stem Cell Derivatives

**DOI:** 10.3389/fimmu.2017.00645

**Published:** 2017-06-02

**Authors:** Xin Liu, Wenjuan Li, Xuemei Fu, Yang Xu

**Affiliations:** ^1^Center for Regenerative and Translational Medicine, Guangdong Provincial Academy of Chinese Medical Sciences, The Second Affiliated Hospital of Guangzhou University of Chinese Medicine, Guangzhou, China; ^2^Division of Biological Sciences, University of California, San Diego, La Jolla, CA, United States; ^3^The Eighth Affiliated Hospital of Sun Yat-sen University, Shenzhen, China

**Keywords:** embryonic stem cells, induced pluripotent stem cells, cell therapy, allogeneic immune rejection, immunogenicity, immune tolerance

## Abstract

Human embryonic stem cells (hESCs) can undergo unlimited self-renewal and differentiate into all cell types in human body, and therefore hold great potential for cell therapy of currently incurable diseases including neural degenerative diseases, heart failure, and macular degeneration. This potential is further underscored by the promising safety and efficacy data from the ongoing clinical trials of hESC-based therapy of macular degeneration. However, one main challenge for the clinical application of hESC-based therapy is the allogeneic immune rejection of hESC-derived cells by the recipient. The breakthrough of the technology to generate autologous-induced pluripotent stem cells (iPSCs) by nuclear reprogramming of patient’s somatic cells raised the possibility that autologous iPSC-derived cells can be transplanted into the patients without the concern of immune rejection. However, accumulating data indicate that certain iPSC-derived cells can be immunogenic. In addition, the genomic instability associated with iPSCs raises additional safety concern to use iPSC-derived cells in human cell therapy. In this review, we will discuss the mechanism underlying the immunogenicity of the pluripotent stem cells and recent progress in developing immune tolerance strategies of human pluripotent stem cell (hPSC)-derived allografts. The successful development of safe and effective immune tolerance strategy will greatly facilitate the clinical development of hPSC-based cell therapy.

## The Promise of Pluripotent Stem Cell (PSC)-Derived Cells in Cell Therapy of Major Human Diseases

The successful establishment of human embryonic stem cells (hESCs) from the inner cell mass of the human blastocyts has opened up a new era in regenerative medicine (1). Significant progress has been achieved in developing protocols to differentiate hESCs into various lineages of biologically active cells such as neural cells, pancreatic β cells, retinal pigmented epithelial (RPE) cells, cardiomyocytes, and hepatocytes (2). Three of such hESC-derived cells have entered clinical trial to treat macular degeneration, spinal cord injury, and type 1 diabetes (3). In addition, these clinical trials have provided promising data on the safety and efficacy of hESC-based cell therapy of macular degeneration, further supporting the great potential of the clinical application of hESC (4–6). However, one of the main bottlenecks that hinder the clinical development of hESC-based therapy is the allogeneic immune rejection by the recipient. While current immune suppression regimen can effectively prevent allogeneic immune rejection, the persistent use of immune suppressants is toxic to patients, and can greatly increase the risk of infection and cancer, especially in patients infected with cytomegalovirus and the herpes viruses (7). In addition, immune suppressants are toxic to some of the hESC-derived progenitor cells (8).

The development of induced pluripotent stem cells (iPSCs) by reprogramming somatic cells into pluripotent state with defined transcriptional factors has provided another source of PSC for human cell therapy (9–12). When compared with hESC-based cell therapy, iPSCs were thought to provide certain advantages, including the lack of ethic concerns and immune rejection by the recipient (13). However, recent studies have demonstrated the increased genomic instability, epigenetic abnormality, and immunogenicity of iPSCs, raising safety concerns of iPSC-based cell therapy (14, 15). Therefore, to improve the feasibility of iPSC-based therapy, it is important to understand the underlying reprogramming mechanism and develop strategies to minimize these safety concerns.

## The Immune Responses to Allogeneic ESCs and Their Derivatives

The polymorphic major histocompatibility complex (MHC) molecules are distinct between individuals, leading to the massive T cell-mediated immune response and rejection of the transplanted allogeneic cells (16). There are two classes of MHC molecules, MHC class I molecules that are expressed on most of the nucleated cells and MHC class II molecules that are expressed primarily on antigen presenting cells (16). MHC class I molecules are required to activate CD8^+^ cytotoxic T cells that can destroy the target cells, and MHC class II molecules are required to activate CD4^+^ helper T cells that are critical to activate other immune cells including cytotoxic T cells (16). In addition, certain cell types lacking the expression of MHC class I molecules such as stem cells and cancer cells are rejected by natural killer (NK) cells (17).

While mouse ESCs do not express detectable MHC Class I and Class II molecules (18, 19), hESCs express MHC Class I molecules but not MHC Class II molecules, and hESCs cannot directly activate T cells *in vitro* and *in vivo* (20–23). However, hESCs can be immune rejected by various mechanisms after transplantation. First, allogeneic NK cells can eliminate mouse and human ESCs *in vitro* (24, 25). Second, while ESCs do not directly activate allogeneic T cell through the interaction between allogeneic MHC molecules and TCR, ESCs express immunogenic antigens such as MHC Class I molecules and Oct4 that can indirectly activate T cells through antigen presenting cells (22, 23, 26). Third, after transplantation, both mouse and human ESCs cannot maintain the pluripotent state and will undergo spontaneous differentiation into various cell types that express MHC molecules, leading to robust T-dependent allogeneic rejection (27, 28). Therefore, hESCs and their derivatives will be immune rejected when transplanted into allogeneic recipients.

While it has been suggested that ESCs might have immune modulatory functions in treating certain diseases such as myocardial infarction (29), ESCs pose a serious teratoma risk after transplantation and thus are unlikely to be used directly in therapy. Therefore, the major effort should be devoted to understanding the allogeneic immune responses to hESC-derived cells that have no teratoma risk. There has been limited progress in this area of research due to the lack of robust animal models to study the human immune responses to hESC-derived cells. Recent application of humanized mice reconstituted with functional human immune system, which are generated by transplanting human fetal thymus and CD34^+^ fetal liver cells, has made this research more feasible (30). In support of this notion, recent studies demonstrated that the humanized mice could mount vigorous allogeneic immune rejection of hESC-derived cells (31).

## The Immunogenicity of iPSCs and Their Derivatives

The breakthrough of induced pluripotency to reprogram somatic cells from patients into PSC has raised the possibility that the cells derived from patient-specific iPSCs are autologous and thus will not be immune rejected by the patient (32). However, hiPSCs can be rejected by allogeneic and autologous NK cells (33). In addition, accumulating data have demonstrated that cells derived from iPSCs can be immunogenic to the autologous immune system. Using an inbred C57/BL6 (B6) transplantation mouse model, Zhao et al. (27) demonstrated that cells derived from B6 iPSCs can activate syngeneic T-dependent immune responses due to the deregulated expression of immunogenic proteins such as the tumor antigen Hormad1. This conclusion is supported by a following study, which also demonstrated that the endothelial cells derived from B6 iPSCs are immune tolerated by B6 mice (34). However, the iPSC-derived endothelial cells express high levels of immune suppressive cytokines such as IL-10 and are immune rejected in B6 mice when treated with anti-IL-10 antibody, supporting the notion that these iPSC-derived endothelial cells are intrinsically immunogenic (34). In addition, cardiomyocytes derived from B6 iPSCs are highly immunogenic when transplanted into the B6 mice subcutaneously (35). In the same study, the authors derived GFP^+^ skin tissue from B6 chimeric mice generated by injecting GFP-expressing B6 iPSCs into B6 blastocyts and found no immune rejection when the GFP^+^ skin tissue was grafted to new B6 mice. However, these B6 iPSC-derived skin tissues have already been preselected by the B6 immune system in the chimeric mice, and thus the skin graft will not be rejected by B6 immune system when transplanted onto new B6 mice. In support of this notion, GFP is a foreign protein and immunogenic to B6 mice when expressed in the skin, and GFP-expressing skin tissues of GFP transgenic B6 mice are immune rejected when grafted to the B6 mice (36–38).

Several recent studies have argued that iPSC-derived cells are not immunogenic to the isogenic or autologous immune system. By transplanting cells derived from B6 iPSCs under the kidney capsule of B6 mice, Guha et al. (39) concluded that various lineages of iPSC-derived cells exhibit no immunogenicity. In addition, the transplantation of iPSC-derived neural cells in the brain of autologous monkeys leads to minimum immune response (40). To resolve this apparent discrepancy, a recent study showed that the lack of immunogenicity of B6-derived iPSCs transplanted under the kidney capsule is due to the absence of functional antigen presenting cells in the kidney, and the cotransplantation of B6 iPSC-derived cells with functional B6 dendritic cells under the kidney capsule leads to robust immune rejection (41). Therefore, the presence of functional antigen presenting cells at the transplantation site is required to reveal the immunogenicity of iPSC-derived cells. In further support of the conclusion that iPSC-derived cells can be immunogenic, using a humanized mouse model reconstituted with human immune system, Zhao et al. demonstrated the differential immunogenicity of human iPSC-derived cells. In this context, iPSC-derived smooth muscle cells are highly immunogenic to the autologous immune system due to the deregulated expression of immunogenic proteins, but iPSC-derived RPE cells are not immunogenic to the autologous immune system even when transplanted into smooth muscle (28). Importantly, the lack of immunogenicity of iPSC-derived RPE cells has been recapitulated in the only one treated patient in the clinical trial to use autologous iPSC-derived RPE cells to treat macular degeneration (42).

There are several mechanisms to explain the immunogenicity of iPSCs. First, compared to ESCs, iPSCs are epigenetically abnormal and inherited epigenetic signature of parental cells (43–45). The epigenetic abnormalities could explain the abnormal expression of immunogenic proteins expressed during the differentiation of iPSCs but not ESCs (27, 28). The impact of this epigenetic mechanism on the deregulated expression of immunogenic proteins could be cell lineage specific as observed in the differentiation of hiPSCs into RPEs and smooth muscle cells (28). Second, somatic coding mutations are detected in all iPSC lines tested, and the protein coding mutations can create new immunogenic determinants like the tumor antigens developed in cancer cells (46, 47). Third, the genomic translocation detected in the iPSCs will create fusion proteins and new immunogenic determinants (14). Therefore, in addition to the immunogenicity of iPSC-derived cells, a more serious safety concern of the iPSC-based cell therapy is the genomic instability of iPSCs that can greatly increase cancer risk (14). In support of this notion, the first clinical trials of iPSC-based cell therapy was terminated after the enrollment of the first patient due to the discovery of coding mutations in cancer related genes in the iPSCs reprogrammed from the somatic cells of the second patient (42). This cancer concern poses a serious bottleneck for developing individualized cell therapy using patients’ own iPSC. Instead, future effort will be devoted to establish genetically stable iPSC cell bank, and the iPSC line with the best match to the patient’s HLA will be used for cell therapy. In this context, the clinic development of allogeneic iPSC will face the same challenge of immune rejection as hESCs.

## Strategies to Protect Allogeneic Human Pluripotent Stem Cell (hPSC)-Derived Cells from Immune Rejection

While immune suppressant regimen can effectively suppress allogeneic immune responses, the persistent use of immune suppressants will significantly increase the risk of infection and cancer, especially in the majority of the population with Cytomegalovirus infection (7). In addition, the immune suppressants are toxic to some hESC-derived cells such as neural progenitor cells (48). Therefore, to realize the potential of hESC-based therapy, it is important to develop safe and effective immune tolerance strategy of allogeneic hESC-derived cells.

Allogeneic immune rejection is primarily mediated by T cell-dependent immune responses. Previous studies have demonstrated that standard immunosuppressive drug regimens can significantly reduce the immune response to prolong the survival of hESC-derived xenografts but cannot eventually prevent immune rejection (49, 50). Based on our knowledge of the pathways critical for T-cell activation, it is possible to induce immune tolerance of hESC-derived allogeneic cells by disrupting the T cell costimulatory pathways, such as CD28-CD80/CD86 and CD40–CD40L pathways (51, 52). PD-L1 also plays a central role in maintaining T cell anergy and preventing autoimmunity (51). PD-L1 (B7-H1, CD274) interacts with programmed cell death 1 (PD-1), which is an inhibitory receptor expressed on activated T cells, NK cells, and B cells, and this interaction leads to negative regulation of lymphocyte activation (53), and PD-1 is required for the induction of T-cell tolerance by dendritic cells (54). Therefore, it is possible to develop immune tolerance strategy by modulating these costimulatory and inhibitory pathways.

A recent study has shown that treatment of mice with cytotoxic T lymphocyte antigen 4-Ig (CTLA4-Ig), which binds to the B7 family of costimulatory molecules CD80 and CD86 with higher affinity and avidity than CD28, and anti-CD40L blocking CD40-CD40L pathway not only prolonged hESC-derived pancreatic endoderm graft survival but also resulted in the establishment of immune tolerance of the xeno-immune system (55). A similar study also showed that the blockade of costimulatory pathways with antibodies significantly prolonged the engraftment of cells derived from human ESCs and iPSCs in mice without inhibiting the ability of the recipient to respond to other foreign antigens (56). However, in contrast to the naive T cells of a mouse model, up to 50% of alloreactive T cells in a human receiving transplants may display a memory phenotype (57, 58), and the allogeneic immune tolerance of mouse immune system can be achieved more easily than human immune system. Therefore, the immune tolerance strategy developed in mouse models must be re-evaluated in the context of human immune system.

To address this bottleneck, recent studies have employed humanized mice reconstituted with human immune system to study human immune responses to cells derived from hPSCs (28, 31, 51, 53, 54, 59). The human immune system of humanized mice can robustly reject allogeneic cells derived from human embryonic stem cells (hESCs), and thus can be used to vigorously test immune tolerance strategy (31). Using humanized mice, it was demonstrated that the expression of CTLA4-Ig and PD-L1 in the cells derived from genetically modified hESCs can effectively protect these cells from allogeneic human immune responses without inducing systemic immune suppression (31). Neither the expression of CTLA4-Ig nor PD-L1 alone is sufficient to provide immune protection, consistent with human clinical data (31, 58). Because the CTLA4-Ig/PD-L1-expressing cells are immune evasive, they are susceptible to infection and cancer. To minimize the risk, the co-expression of the suicidal thymidine kinase gene in these cells enables the efficient elimination of the graft with FDA-approved TK-targeting drug (59). These studies supported the feasibility to develop safe and effective strategy to protect hESC-derived allografts from immune rejection.

In addition to the modulation of the T cell costimulatory and inhibitory pathways, other strategies have been explored to confer immune protection of allogeneic hESC-derived allografts. One such attempt is to reduce the immunogenicity of hESC-derived cells by disrupting the expression of MHC molecules that elicit allogeneic T-cell responses. For example, knockdown of both HLA class I and class II in hESC could provoke T-cell ignorance *in vitro* and protection from xenogeneic immune rejection *in vivo* (60–62). Disruption of MHC class II transactivator in hESCs leads to the silencing of HLA class II expression in differentiated cells, such genetic modification could confer protection from the allogeneic helper T cells (63, 64). While the immune evasive properties of these cells lacking HLA I and II molecules have been confirmed *in vitro* and in mice, they remain to be evaluated in the context of a human immune system.

## Conclusion

Despite their potential in curing major diseases and the encouraging clinical trial data, one of the key challenges for hPSC-based therapy is the immune rejection by the recipient. With extensive data of the transplantation immunology in mice and the accumulating data on the human immune responses to hESC-derived allografts in humanized mice, it has become apparently feasible to develop safe and effective strategies to induce graft-specific immune tolerance in the near future. Such achievement will greatly facilitate the clinic development of hPSC-based therapy.

## Author Contributions

All authors listed have made substantial, direct, and intellectual contribution to the work and approved it for publication.

## Conflict of Interest Statement

The authors declare that the research was conducted in the absence of any commercial or financial relationships that could be constructed as a potential conflict of interest.

## References

[1] Thomson JA Itskovitz-Eldor J Shapiro SS Waknitz MA Swiergiel JJ Marshall VS Embryonic stem cell lines derived from human blastocysts. Science (1998) 282:1145–7. 10.1126/science.282.5391.1145 9804556

[2] Fu X Xu Y. Self-renewal and scalability of human embryonic stem cells for human therapy. Regen Med (2011) 6:327–34. 10.2217/rme.11.18 21548738

[3] Fu X Cui K Yi Q Yu L Xu Y. DNA repair mechanisms in embryonic stem cells. Cell Mol Life Sci (2017) 74:487–93. 10.1007/s00018-016-2358-z 27614628 PMC11107665

[4] Song WK Park KM Kim HJ Lee JH Choi J Chong SY Treatment of macular degeneration using embryonic stem cell-derived retinal pigment epithelium: preliminary results in Asian patients. Stem Cell Reports (2015) 4:860–72. 10.1016/j.stemcr.2015.04.005 25937371 PMC4437471

[5] Schwartz SD Hubschman JP Heilwell G Franco-Cardenas V Pan CK Ostrick RM Embryonic stem cell trials for macular degeneration: a preliminary report. Lancet (2012) 379:713–20. 10.1016/S0140-6736(12)60028-2 22281388

[6] Schwartz SD Regillo CD Lam BL Eliott D Rosenfeld PJ Gregori NZ Human embryonic stem cell-derived retinal pigment epithelium in patients with age-related macular degeneration and Stargardt’s macular dystrophy: follow-up of two open-label phase 1/2 studies. Lancet (2015) 385:509–16. 10.1016/S0140-6736(14)61376-3 25458728

[7] Fishman JA. Overview: cytomegalovirus and the herpesviruses in transplantation. Am J Transplant (2013) 13:1–8. 10.1111/ajt.12002 23347210

[8] Herberts CA Kwa MS Hermsen HP Risk factors in the development of stem cell therapy. J Transl Med (2011) 9:29–29. 10.1186/1479-5876-9-29 21418664 PMC3070641

[9] Takahashi K Yamanaka S. Induction of pluripotent stem cells from mouse embryonic and adult fibroblast cultures by defined factors. Cell (2006) 126:663–76. 10.1016/j.cell.2006.07.024 16904174

[10] Takahashi K Tanabe K Ohnuki M Narita M Ichisaka T Tomoda K Induction of pluripotent stem cells from adult human fibroblasts by defined factors. Cell (2007) 131:861–72. 10.1016/j.cell.2007.11.019 18035408

[11] Yu J Vodyanik MA Smuga-Otto K Antosiewicz-Bourget J Frane JL Tian S Induced pluripotent stem cell lines derived from human somatic cells. Science (2007) 318:1917–20. 10.1126/science.1151526 18029452

[12] Park IH Zhao R West JA Yabuuchi A Huo H Ince TA Reprogramming of human somatic cells to pluripotency with defined factors. Nature (2008) 451:141–6. 10.1038/nature06534 18157115

[13] Ilic D Ogilvie C Concise review: human embryonic stem cells—what have we done? what are we doing? where are we going? Stem Cells (2017) 35:17–25. 10.1002/stem.2450 27350255

[14] Yoshihara M Hayashizaki Y Murakawa Y Genomic instability of iPSCs: challenges towards their clinical applications. Stem Cell Rev Rep (2017) 13:7–16. 10.1007/s12015-016-9680-6 PMC5346115 27592701

[15] Sebban S Buganim Y. Nuclear reprogramming by defined factors: quantity versus quality. Trends Cell Biol (2016) 26:65–75. 10.1016/j.tcb.2015.08.006 26437595

[16] Marino J Paster J Benichou G. Allorecognition by T lymphocytes and allograft rejection. Front Immunol (2016) 7:582. 10.3389/fimmu.2016.00582 28018349 PMC5155009

[17] Chidgey AP Boyd RL. Immune privilege for stem cells: not as simple as it looked. Cell Stem Cell (2008) 3:357–8. 10.1016/j.stem.2008.09.011 18940724

[18] Boyd AS Wood KJ Variation in MHC expression between undifferentiated mouse ES cells and ES cell derived insulin-producing cell clusters. Transplantation (2009) 87:1300–4. 10.1097/TP.0b013e3181a19421 19424028 PMC2796795

[19] Jaffe L Robertson EJ Bikoff EK. Distinct patterns of expression of MHC class I and beta 2-microglobulin transcripts at early stages of mouse development. J Immunol (1991) 147:2740. 1918988

[20] English K Wood KJ. Immunogenicity of embryonic stem cell-derived progenitors after transplantation. Curr Opin Organ Transplant (2011) 16:90–5. 10.1097/MOT.0b013e3283424faa 21150615

[21] Draper JS Pigott C Thomson JA Andrews PW Surface antigens of human embryonic stem cells: changes upon differentiation in culture*. J Anat (2002) 200:249–58. 10.1046/j.1469-7580.2002.00030.x 12033729 PMC1570685

[22] Li L Baroja ML Majumdar A Chadwick K Rouleau A Gallacher L Human embryonic stem cells possess immune-privileged properties. Stem Cells (2004) 22:448–56. 10.1634/stemcells.22-4-448 15277692

[23] Drukker M Katchman H Katz G Even-Tov Friedman S Shezen E Hornstein E Human embryonic stem cells and their differentiated derivatives are less susceptible to immune rejection than adult cells. Stem Cells (2006) 24:221–9. 10.1634/stemcells.2005-0188 16109762

[24] Dressel R Nolte J Elsner L Novota P Guan K Streckfuss-Bömeke K Pluripotent stem cells are highly susceptible targets for syngeneic, allogeneic, and xenogeneic natural killer cells. FASEB J (2010) 24:2164–77. 10.1096/fj.09-134957 20145206

[25] Perez-Cunningham J Ames E Smith RC Peter AK Naidu R Nolta JA Natural killer cell subsets differentially reject embryonic stem cells based on licensing. Transplantation (2014) 97:992–8. 10.1097/TP.0000000000000063 24704665

[26] Dhodapkar KM Feldman D Matthews P Radfar S Pickering R Turkula S Natural immunity to pluripotency antigen OCT4 in humans. Proc Natl Acad Sci U S A (2010) 107:8718–23. 10.1073/pnas.0915086107 20404147 PMC2889362

[27] Zhao T Zhang ZN Rong Z Xu Y. Immunogenicity of induced pluripotent stem cells. Nature (2011) 474:212–5. 10.1038/nature10135 21572395

[28] Zhao TB Zhang ZN Westenskow PD Todorova D Hu Z Lin TX Humanized mice reveal differential immunogenicity of cells derived from autologous induced pluripotent stem cells. Cell Stem Cell (2015) 17:353–9. 10.1016/j.stem.2015.07.021 26299572 PMC9721102

[29] Yuan X Zhang H Wei YJ Hu SS Embryonic stem cell transplantation for the treatment of myocardial infarction: immune privilege or rejection. Transpl Immunol (2007) 18:88–93. 10.1016/j.trim.2007.05.003 18005850

[30] Shultz LD Brehm MA Garcia-Martinez JV Greiner DL. Humanized mice for immune system investigation: progress, promise and challenges. Nat Rev Immunol (2012) 12:786–98. 10.1038/nri3311 23059428 PMC3749872

[31] Rong ZL Wang MY Hu Z Stradner M Zhu SY Kong HJ An effective approach to prevent immune rejection of human ESC-derived allografts. Cell Stem Cell (2014) 14:121–30. 10.1016/j.stem.2013.11.014 24388175 PMC4023958

[32] Yamanaka S. Strategies and new developments in the generation of patient-specific pluripotent stem cells. Cell Stem Cell (2007) 1:39–49. 10.1016/j.stem.2007.05.012 18371333

[33] Kruse V Hamann C Monecke S Cyganek L Elsner L Hübscher D Human induced pluripotent stem cells are targets for allogeneic and autologous natural killer (NK) cells and killing is partly mediated by the activating NK receptor DNAM-1. PLoS One (2015) 10:e0125544. 10.1371/journal.pone.0125544 25950680 PMC4423859

[34] de Almeida PE Meyer EH Kooreman NG Diecke S Dey D Sanchez-Freire V Transplanted terminally differentiated induced pluripotent stem cells are accepted by immune mechanisms similar to self-tolerance. Nat Commun (2014) 5:3903. 10.1038/ncomms4903 24875164 PMC4075468

[35] Araki R Uda M Hoki Y Sunayama M Nakamura M Ando S Negligible immunogenicity of terminally differentiated cells derived from induced pluripotent or embryonic stem cells. Nature (2013) 494:100–4. 10.1038/nature11807 23302801

[36] Andersson G Illigens BM Johnson KW Calderhead D LeGuern C Benichou G Nonmyeloablative conditioning is sufficient to allow engraftment of EGFP-expressing bone marrow and subsequent acceptance of EGFP-transgenic skin grafts in mice. Blood (2003) 101:4305. 10.1182/blood-2002-06-1649 12576326

[37] Andersson G Denaro M Johnson K Morgan P Sullivan A Houser S Engraftment of retroviral EGFP-transduced bone marrow in mice prevents rejection of EGFP-transgenic skin grafts. Mol Ther (2003) 8:385–91. 10.1016/S1525-0016(03)00210-7 12946311

[38] Han WG Unger WW Wauben MH Identification of the immunodominant CTL epitope of EGFP in C57BL/6 mice. Gene Ther (2008) 15:700–1. 10.1038/sj.gt.3303104 18288211

[39] Guha P Morgan JW Mostoslavsky G Rodrigues NP Boyd AS Lack of immune response to differentiated cells derived from syngeneic induced pluripotent stem cells. Cell Stem Cell (2013) 12:407–12. 10.1016/j.stem.2013.01.006 23352605

[40] Morizane A Doi D Kikuchi T Okita K Hotta A Kawasaki T Direct comparison of autologous and allogeneic transplantation of iPSC-derived neural cells in the brain of a nonhuman primate. Stem Cell Reports (2013) 1:283–92. 10.1016/j.stemcr.2013.08.007 24319664 PMC3849265

[41] Todorova D Kim J Hamzeinejad S He J Xu Y. Brief report: immune microenvironment determines the immunogenicity of induced pluripotent stem cell derivatives. Stem Cells (2016) 34:510–5. 10.1002/stem.2227 26439188

[42] Mandai M Watanabe A Kurimoto Y Hirami Y Morinaga C Daimon T Autologous induced stem-cell–derived retinal cells for macular degeneration. N Engl J Med (2017) 376:1038–46. 10.1056/NEJMoa1608368 28296613

[43] Lister R Pelizzola M Kida YS Hawkins RD Nery JR Hon G Hotspots of aberrant epigenomic reprogramming in human induced pluripotent stem cells. Nature (2011) 471:68–73. 10.1038/nature09798 21289626 PMC3100360

[44] Chin MH Mason MJ Xie W Volinia S Singer M Peterson C Induced pluripotent stem cells and embryonic stem cells are distinguished by gene expression signatures. Cell Stem Cell (2009) 5:111–23. 10.1016/j.stem.2009.06.008 19570518 PMC3448781

[45] Ruiz S Diep D Gore A Panopoulos AD Montserrat N Plongthongkum N Identification of a specific reprogramming-associated epigenetic signature in human induced pluripotent stem cells. Proc Natl Acad Sci U S A (2012) 109:16196–201. 10.1073/pnas.1202352109 22991473 PMC3479609

[46] Gore A Li Z Fung HL Young JE Agarwal S Antosiewicz-Bourget J Somatic coding mutations in human induced pluripotent stem cells. Nature (2011) 471:63–7. 10.1038/nature09805 21368825 PMC3074107

[47] Li Z Lu H Yang W Yong J Zhang ZN Zhang K Mouse SCNT ESCs have lower somatic mutation load than syngeneic iPSCs. Stem Cell Reports (2014) 2:399–405. 10.1016/j.stemcr.2014.02.005 24749065 PMC3986627

[48] Preynat-Seauve O de Rham C Tirefort D Ferrari-Lacraz S Krause KH Villard J. Neural progenitors derived from human embryonic stem cells are targeted by allogeneic T and natural killer cells. J Cell Mol Med (2009) 13:3556–69. 10.1111/j.1582-4934.2009.00746.x 19320778 PMC4516508

[49] Swijnenburg RJ Tanaka M Vogel H Baker J Kofidis T Gunawan F Embryonic stem cell immunogenicity increases upon differentiation after transplantation into ischemic myocardium. Circulation (2005) 112:I166–72. 10.1161/CIRCULATIONAHA.104.525824 16159810

[50] Swijnenburg RJ Schrepfer S Govaert JA Cao F Ransohoff K Sheikh AY Immunosuppressive therapy mitigates immunological rejection of human embryonic stem cell xenografts. Proc Natl Acad Sci U S A (2008) 105:12991–6. 10.1073/pnas.0805802105 18728188 PMC2529073

[51] Fife BT Bluestone JA. Control of peripheral T-cell tolerance and autoimmunity via the CTLA-4 and PD-1 pathways. Immunol Rev (2008) 224:166–82. 10.1111/j.1600-065X.2008.00662.x 18759926

[52] Francisco LM Sage PT Sharpe AH. The PD-1 pathway in tolerance and autoimmunity. Immunol Rev (2010) 236:219–42. 10.1111/j.1600-065X.2010.00923.x 20636820 PMC2919275

[53] Keir ME Butte MJ Freeman GJ Sharpe AH. PD-1 and its ligands in tolerance and immunity. Annu Rev Immunol (2008) 26:677–704. 10.1146/annurev.immunol.26.021607.090331 18173375 PMC10637733

[54] Raker VK Domogalla MP Steinbrink K Tolerogenic dendritic cells for regulatory T cell induction in man. Front Immunol (2015) 6:569 10.3389/fimmu.2015.00569 26617604 PMC4638142

[55] Szot GL Yadav M Lang J Kroon E Kerr J Kadoya K Tolerance induction and reversal of diabetes in mice transplanted with human embryonic stem cell-derived pancreatic endoderm. Cell Stem Cell (2015) 16:148–57. 10.1016/j.stem.2014.12.001 25533131

[56] Pearl JI Lee AS Leveson-Gower DB Sun N Ghosh Z Lan F Short-term immunosuppression promotes engraftment of embryonic and induced pluripotent stem cells. Cell Stem Cell (2010) 8:309–17. 10.1016/j.stem.2011.01.012 PMC3061351 21362570

[57] Alegre ML Florquin S Goldman M. Cellular mechanisms underlying acute graft rejection: time for reassessment. Curr Opin Immunol (2007) 19:563–8. 10.1016/j.coi.2007.07.019 17720467

[58] Valujskikh A. The challenge of inhibiting alloreactive T-cell memory. Am J Transplant (2006) 6:647–51. 10.1111/j.1600-6143.2005.01215.x 16539619

[59] He J Rong Z Fu X Xu Y. A safety checkpoint to eliminate cancer risk of the immune evasive cells derived from human embryonic stem cells. Stem Cells (2017) 35:1154–61. 10.1002/stem.2568 28090751

[60] Deuse T Seifert M Tyan D Tsao PS Hua X Velden J Immunobiology of naïve and genetically modified HLA-class-I-knockdown human embryonic stem cells. J Cell Sci (2011) 124:3029. 10.1242/jcs.104125 21878509

[61] Deuse T Seifert M Phillips N Fire A Tyan D Kay M Human leukocyte antigen I knockdown human embryonic stem cells induce host ignorance and achieve prolonged xenogeneic survival. Circulation (2011) 124:S3–9. 10.1161/CIRCULATIONAHA.111.020727 21911816

[62] Lu P Chen J He L Ren J Chen H Rao L Generating hypoimmunogenic human embryonic stem cells by the disruption of beta 2-microglobulin. Stem Cell Rev Rep (2013) 9:806–13. 10.1007/s12015-013-9457-0 23934228

[63] Chen H Li Y Lin X Cui D Cui C Li H Functional disruption of human leukocyte antigen II in human embryonic stem cell. Biol Res (2015) 48:59. 10.1186/s40659-015-0051-6 26506955 PMC4624597

[64] Zheng D Wang X Xu RH. Concise review: one stone for multiple birds: generating universally compatible human embryonic stem cells. Stem Cells (2016) 34:2269–75. 10.1002/stem.2407 27251112

